# Fungal‐Bacterial Dysbiosis in IBD: Microbial Biomarkers of Disease Activity

**DOI:** 10.1002/mbo3.70088

**Published:** 2025-11-04

**Authors:** Elisa Arribas‐Rodríguez, María García‐Pizarro, Ángel De Prado, María Santiago‐Carretero, Marta Hernández, Benito Velayos Jiménez, Javier García‐Alonso, Jesús Barrio, José María Eiros, Eduardo Arranz, David Bernardo, Luis Fernández‐Salazar, Sara Cuesta‐Sancho

**Affiliations:** ^1^ Mucosal Immunology Lab. Instituto de Biomedicina y Genética Molecular de Valladolid (IBGM, Universidad de Valladolid‐CSIC) Valladolid Spain; ^2^ Servicio de Gastroenterología Hospital Universitario Río Hortega Valladolid Spain; ^3^ Area of Microbiology, Faculty of Medicine Universidad de Valladolid Valladolid Spain; ^4^ National Influenza Centre Valladolid Spain; ^5^ Gastroenterology Department Hospital Clínico Universitario (SACYL) Valladolid Spain; ^6^ Centro de Investigación Biomédica en Red de Enfermedades Infecciosas (CIBERINFECC) Madrid Spain; ^7^ Microbiology Unit Hospital Universitario Río Hortega Valladolid Spain; ^8^ Microbiology Unit Hospital Clínico Universitario de Valladolid Valladolid Spain; ^9^ Department of Medicine, Dermatology and Toxicology, Faculty of Medicine Universidad de Valladolid Valladolid Spain

**Keywords:** bacteria, biomarkers, fungi, inflammatory bowel disease, microbiota

## Abstract

Inflammatory bowel disease is associated with complex changes in the intestinal microbiota. While bacterial dysbiosis has been widely studied, the role of fungal communities and their interaction with bacteria remains less explored. This study aims to characterize both bacterial and fungal populations in patients with Crohn's disease and ulcerative colitis, identifying potential microbial biomarkers and inter‐kingdom interactions related to disease activity. We analysed paired intestinal tissue and faecal samples from patients with inflammatory bowel disease. Bacterial, fungal and viral composition were assessed by 16S, ITS DNA and shotgun sequencing, respectively. Bioinformatics analyses were done using Qiime2 and R studio platforms. PERMANOVA and ANOSIM analyses revealed significant compositional differences between faecal and mucosal samples in most groups. Several genera were consistently shared across tissues, while others showed tissue‐ or disease‐specific distributions. Exploratory correlations between bacteria and fungi revealed that *Wallemia* could play a role in balancing the presence of potentially beneficial and pathogenic bacteria. Importantly, *Prevotella* was associated with active disease, *Fusobacterium* with active Crohn's disease, and *Roseburia* with remission in ulcerative colitis, supporting their potential as biomarkers. All these hypotheses should be further tested in future studies. Our findings reveal distinct microbial signatures in inflammatory bowel disease that could promote the intestinal dysbiosis hypotheses perpetuate chronic intestinal inflammation. The identification of faecal biomarkers may complement approaches for noninvasive monitoring of disease activity. These results underscore the need for further research into bacteria‐fungi interactions and their role in gut inflammation.

## Graphical abstract

This study characterizes bacterial, fungal, and viral communities in Crohn's disease and ulcerative colitis using intestinal and faecal samples. Distinct microbial signatures and inter‐kingdom interactions were identified, with taxa such as Prevotella, Fusobacterium, and Roseburia linked to disease activity. Findings highlight potential biomarkers and the need for deeper bacteria–fungi research.

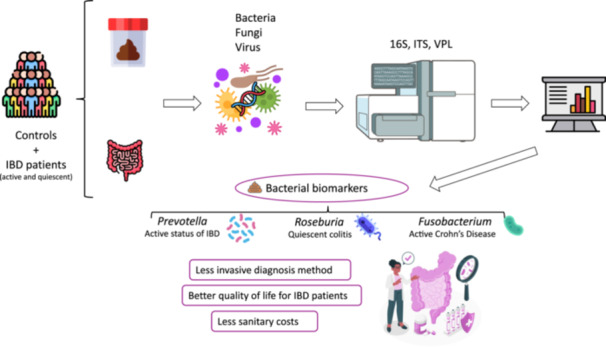

## 1 Introduction

Inflammatory bowel disease (IBD) is a chronic inflammation of the gastrointestinal tract (GIT) mediated by an altered immune response. IBD includes Crohn's disease (CD) and ulcerative colitis (UC), which are differentiated by the type of inflammation, intestinal location, symptoms and associated complications (Gomollón et al. 2017; Magro et al. 2017). Although the aetiology of the disease is not yet known, current evidence suggests that both UC and CD result from inappropriate immune activation directed against intestinal microorganisms (Rogler 2017). The burden of IBD continues to expand, particularly across Western populations, with European cases reaching approximately 3 million individuals (Kaplan 2015).

The immune system can establish a balance between immune responses against pathogens and tolerogenic responses against commensal microbiota (Iebba et al. 2016). In addition to the composition of the intestinal microbiota itself, it is important to highlight that microorganisms generate metabolites, called postbiotics, which have various properties, exert significant immunomodulatory effects. Short chain fatty acids (SCFAs) such as butyrate, propionate and acetate are products of bacterial action on dietary fiber. These metabolites can modulate histone acetylases during immune responses, thereby affecting gene expression (Tian et al. 2024). Notably, IBD patients demonstrate reduced populations of butyrate‐synthesizing bacteria, such as *Roseburia spp (*Imhann et al. 2018 *)*. Similarly, microbial transformation of tryptophan generates indole‐containing bioactive compounds that engage the aryl hydrocarbon receptor—a transcription factor governing inflammatory processes in immune cells, in the case of IBD, tryptophan metabolism is reduced (Schirmer et al. 2019). Moreover, bacterially‐derived secondary bile acids regulate genetic programs controlling immune cell development, cytokine production, and antimicrobial defence mechanisms (Devlin and Fischbach 2015). This production of secondary bile acids is decreased in patients with IBD (Di Ciaula et al. 2023).

Host genetics, environmental exposures, lifestyle patterns, and dietary habits collectively shape gut microbial communities. The transition to a state of dysbiosis can trigger or exacerbate autoimmune and inflammatory responses (Haneishi et al. 2023; Imai et al. 2019; Levy et al. 2017). Amplicon sequencing studies using the 16S ribosomal RNA gene have revealed differences in the mucosal‐associated intestinal microbiota of patients with IBD compared to healthy controls (Gevers et al. 2014), including a depletion of *Firmicutes* (which have an anti‐inflammatory effect) and *Clostridium species*, as well as an increase in the abundance of *Bacteroidetes* and *Proteobacteria*, such as *Bacteroides, Bifidobacterium* and *Lactobacillus*, which are associated with inflammation (Schirmer et al. 2019; Andoh et al. 2011).

Nonbacterial enteric microorganisms, including fungi and viruses, also participate in maintaining gut equilibrium. Although research examining gastrointestinal fungi and viruses in IBD pathogenesis remains limited, dysbiotic patterns within these microbial communities have been implicated in disease development (Iliev and Cadwell 2021). IBD patients exhibit a higher *Basidiomycota/Ascomycota* ratio, reduced *Saccharomyces cerevisiae* and increased *Candida albicans* compared to controls (Sokol et al. 2017). While diverse fungal species inhabit the human gut, only select species show consistent presence across individuals. The enteric virome represents an emerging research frontier given its potential influence on digestive homeostasis (Virgin 2014). However, the high variability observed among individuals complicates the identification of specific relationships IBD‐virome (Finkbeiner et al. 2008; Minot et al. 2011; Ungaro et al. 2019).

Despite advances in understanding IBD, its pathogenesis remains incompletely defined, and current treatments are far from optimal. Therefore, a comprehensive characterization of enteric microorganisms in different IBD types (UC and CD) and disease states (active vs. quiescent) is needed. The present study aims to analyse the microbial composition of faeces and biopsies from IBD patients and healthy controls. Hence, these results might identify microbial interactions and their potential role in disease pathogenesis. This knowledge could contribute to improved diagnostic and therapeutic strategies.

## 2 Materials and Methods

### 2.1 Biological Samples

Biopsies and stool samples (*n* = 30) were obtained from four groups of patients with IBD: CD—both active and quiescent—, and UC, also active and quiescent. Samples were also collected from individuals attending medical consultation for reasons other than IBD and who have healthy mucosa during colonoscopy and constitute control group. Samples were collected from both the Hospital Clínico Universitario de Valladolid and Hospital Universitario Río Hortega de Valladolid. All participants provided signed informed consent (approval code by the CEIm Area del Salud de Valladolid PI 22‐2869). Information was collected for each patient regarding age and gender, current treatments, as well as any treatments received in the 4 weeks before sample collection (if applicable) including antibiotics (people who had taken antibiotics for at least 3 months before sample collection were discarded). Additionally, disease phenotype in CD (B1 inflammatory; B2 stenosing; B3 penetrating or fistulizing), disease location according to the Montreal classification: CD (L1 ileal; L2 colonic; L3 ileocolonic; L4 upper GIT), UC (E1 proctitis; E2 distal colitis; E3 extensive colitis) any other information that might be relevant for interpreting the results was also collected and it is shown in Supporting Information S2: Table S1.

Faecal samples were obtained from 30 individuals: five controls, six patients with active Crohn's disease, six patients with quiescent Crohn's disease, five patients with active UC and seven patients with quiescent UC. Biopsies were obtained from the ileum of 25 of these 30 patients (five from each group). Information about gender, age and other relevant information about recluted patients is collected in Supporting Information S2: Table S2. Patient 11—from quiescent Crohn's cohort—was excluded from the study due to not meeting the inclusion criteria, as antibiotic use was identified, leaving a final study population of 24 patients.

Stool samples were collected in faecal collection tubes (Canvax Biotech, Valladolid, Spain) during the 24 h before the endoscopy and cryopreserved at −80°C. Following biopsy obtention, they were preserved in ice‐chilled Phosphate‐buffered saline (PBS) (ThermoFisher Scientific, Waltham, USA) and subsequently cryopreserved at −80°C in RNAlater (ThermoFisher Scientific, Waltham, USA).

### 2.2 Biopsies Processing and Sequencing

DNA extraction and sequencing was carried out by Seqplexing (Sequencing Multiplex, Valencia) using an Illumina MiSeq device, paired‐end 2 x 250bp. For bacteria (16S DNA), the primers used for amplification were 16S V1‐V2 Forward Primer 5’‐1: TNANACATGCAAGTCGRRSG; 16S V1‐V2 Forward Primer 5’‐2: TAACACATGCAAGTCRACTYGA and 16S V1‐V2 Reverse Primer 3’: GCTGCCTCCCGTAGGAGT. For fungi (ITS2 region), the primers used were ITS2_F1: GTGARTCATCGAATCTTTG, ITS2_R1: TCCTCCGCTTATTGATATGC and ITS2_R2: GATATGCTTAAGTTCAGCGGGT. Biopsies were also used to study viral composition using a Shotgun approach also by Seqplexing (Sequencing Multiplex, Valencia). As part of their standard workflow, the provider applied quality control procedures, including read filtering and removal of low‐quality sequences. The processed datasets generated by Seqplexing were used for downstream analyses in this study.

### 2.3 Stool Samples Processing and Sequencing

Bacterial DNA was extracted following manufacturer's recommendations for the QIAamp PowerFecal Pro DNA Kit (Qiagen, Venlo, Netherlands). Sample DNA was quantified using a NanoDrop microvolume spectrophotometer (Thermo Fisher, USA) and then stored at −20°C until shipment. Metagenomic sequencing was performed at the University of Valladolid on a MiSeq platform (Illumina, San Diego, CA, USA) using the Nextera XT Index Kit paired‐end (2×300nt) (Illumina, San Diego, CA, USA) for microbiota analysis by amplifying the hypervariable V3 and V4 regions of 16S rDNA gene with specific primers (515F‐806R). As part of their standard workflow, the provider applied quality control procedures, including read filtering and removal of low‐quality sequences. The processed datasets generated were used for downstream analyses in this study.

### 2.4 Bioinformatic Analysis

Raw sequencing reads were processed using Qiime2 (https://qiime2.org/), an open‐source bioinformatics platform designed for the analysis of microbial communities using high‐throughput sequencing data. After assessing the quality of the demultiplexed reads, sequences were denoised using the DADA2 algorithm (Callahan et al. 2016), this process generated a feature table of Amplicon Sequence Variants (ASVs). During this step, forward reads were trimmed by 6 bp from the 5' end and truncated at 240 bp, while reverse reads were trimmed by 7 bp and truncated at 240 bp.

For phylogenetic analysis, sequences were aligned using the MAFT algorithm (Katoh 2002). Taxonomic classification was then assigned to each ASV using a pre‐trained Naive Bayes classifier against the appropriate reference database (SILVA for bacteria and UNITE for fungi). The resulting ASV feature table, taxonomy table and phylogenetic tree were then exported for downstream analysis in R (https://www.r-project.org/). In addition, beta diversity was calculated and plots were generated to help visualize its results.

An exploratory analysis was conducted to visualize the microbial composition. Stack bar plots were generated to visualize the top 15 genus of bacteria and fungi in biopsies and of bacteria in stools. To statistically test for significant differences in the overall community structure (beta diversity), both ANOSIM and PERMANOVA tests were performed. The analyses were structured to compare bacterial communities between sample types (biopsies vs. stools) and to evaluate differences among patient groups for both bacteria and fungi.

Moreover, within‐sample diversity (alpha diversity) was analysed for bacteria (separately for biopsies and stools) and fungi. Shannon and Simpson indices were calculated, and the Kruskal‐Wallis test was used to assess for statistically significant differences in these metrics between patient groups. The results were visualized using boxplots.

Differentially abundant taxa were identified using Linear discriminant analysis Effect Size (LEfSe) (Segata et al. 2011). The analysis was conducted at the genus level, using a logarithmic transformation of the data and a threshold for the Kruskall‐Wallis test of *α* = 0.05. For bacteria samples the comparisons were performed to identify biomarkers associated with the sample type (biopsies vs. Stools). In both bacteria and fungi, comparisons were performed to identify biomarkers associated with the patient group.

Inter‐kingdom associations were investigated by calculating Spearman's rank correlation between the relative abundances of the top 20 bacterial genera and all the detected fungal genera (Akiyama et al. 2024). The results were visualized using a heatmap. The decision to use relative abundances despite the compositional nature of microbiome data was due to the fact that the data set used for this study has a small sample size and is highly sparse. These characteristics, when using the CLR transformation can introduce noise and potential bias, which led to multiple perfect correlations (rho = 1, *p* = 0) that were statistically significant but biologically implausible. Therefore, all results regarding the inter‐kingdom correlations should be viewed as exploratory and be tested in further studies with a bigger sample size and a less sparse data set.

The code used for this project is publicly available in the following Github repository: https://github.com/mariagpms/Microbiome-Analysis.git

## 3 Results

### 3.1 Microbial Diversity in Intestinal Biopsies

#### 3.1.1 Bacterial Diversity and Composition

**Figure 1:**
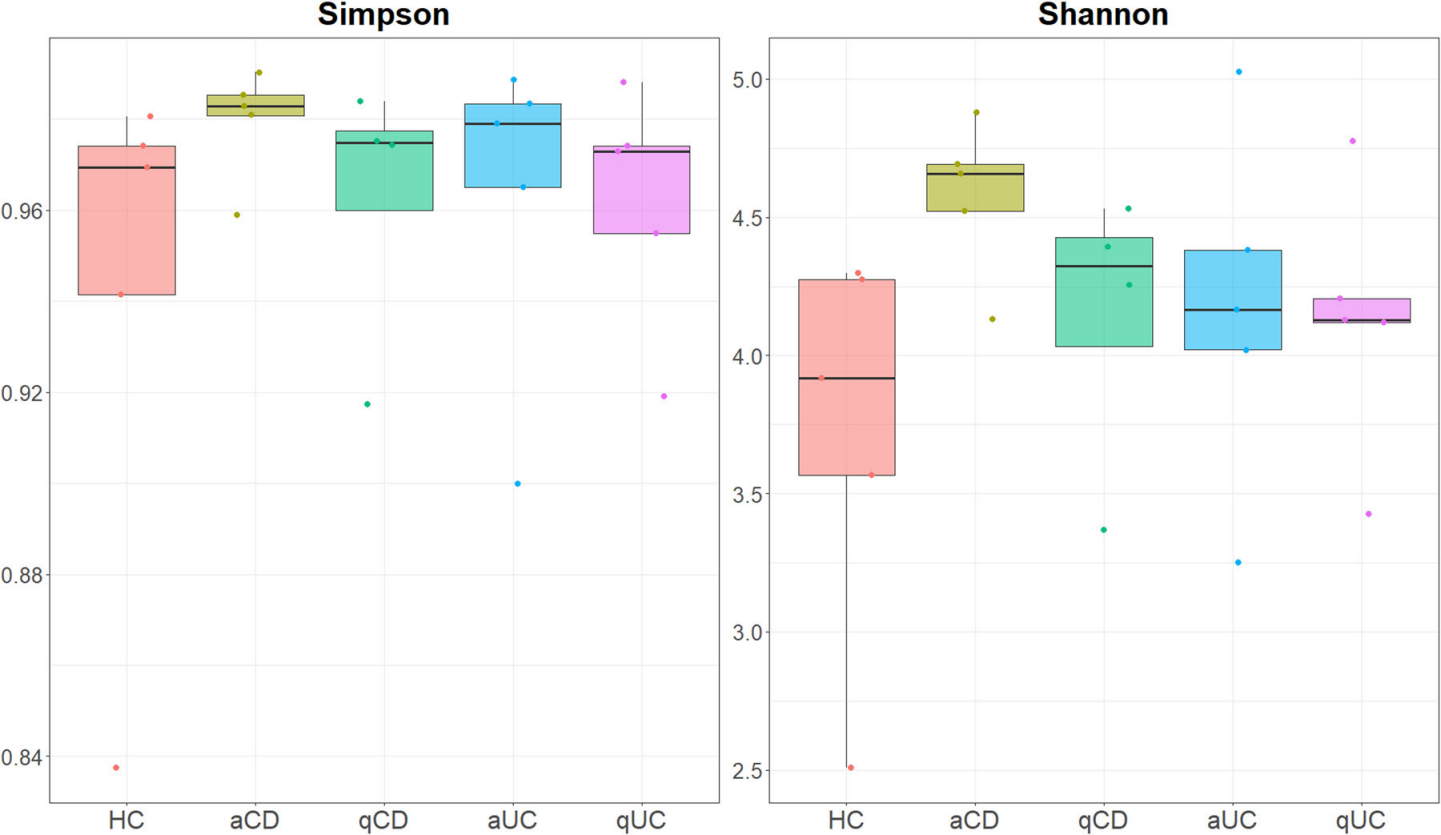
Bacterial alpha diversity in intestinal biopsies. Shannon and Simpson indices of alpha diversity of the bacteria were calculated in intestinal biopsies from the different studied groups: healthy controls (HC), active Crohn's disease (aCD), quiescent Crohn's disease (qCD), active colitis (aUC) and quiescent colitis (qUC). Kruskal Wallis test was then performed. *p*‐value < 0.05 was considered significant.

Bacterial Alpha diversity in intestinal biopsies was analysed using both Shannon and Simpson indices (Figure 1). Even though there were no significant differences, alpha diversity indices revealed distinct microbial patterns. The control group tended to show relatively balanced microbial diversity, with a slight predominance of certain species, although these differences were not statistically significant. In contrast, both active UC and CD groups appeared to have higher microbial diversity with a lower dominance of specific taxa; however, these observations were not supported by significant statistical differences. Notably, the quiescent UC and CD groups tended to exhibit higher diversity and evenness, which may suggest a partial restoration of microbiota composition during remission, though these findings did not reach statistical significance (Figure 1).

Beta diversity analysis revealed no significant differences between groups. Bray‐Curtis, Jaccard, and UniFrac (weighted and unweighted) distance metrics were used to assess community composition. Although no statistically significant differences were detected, variability among individuals was evident, suggesting a heterogeneous microbial landscape irrespective of disease status (Supporting Information S1: Figure S1).

**Figure 2:**
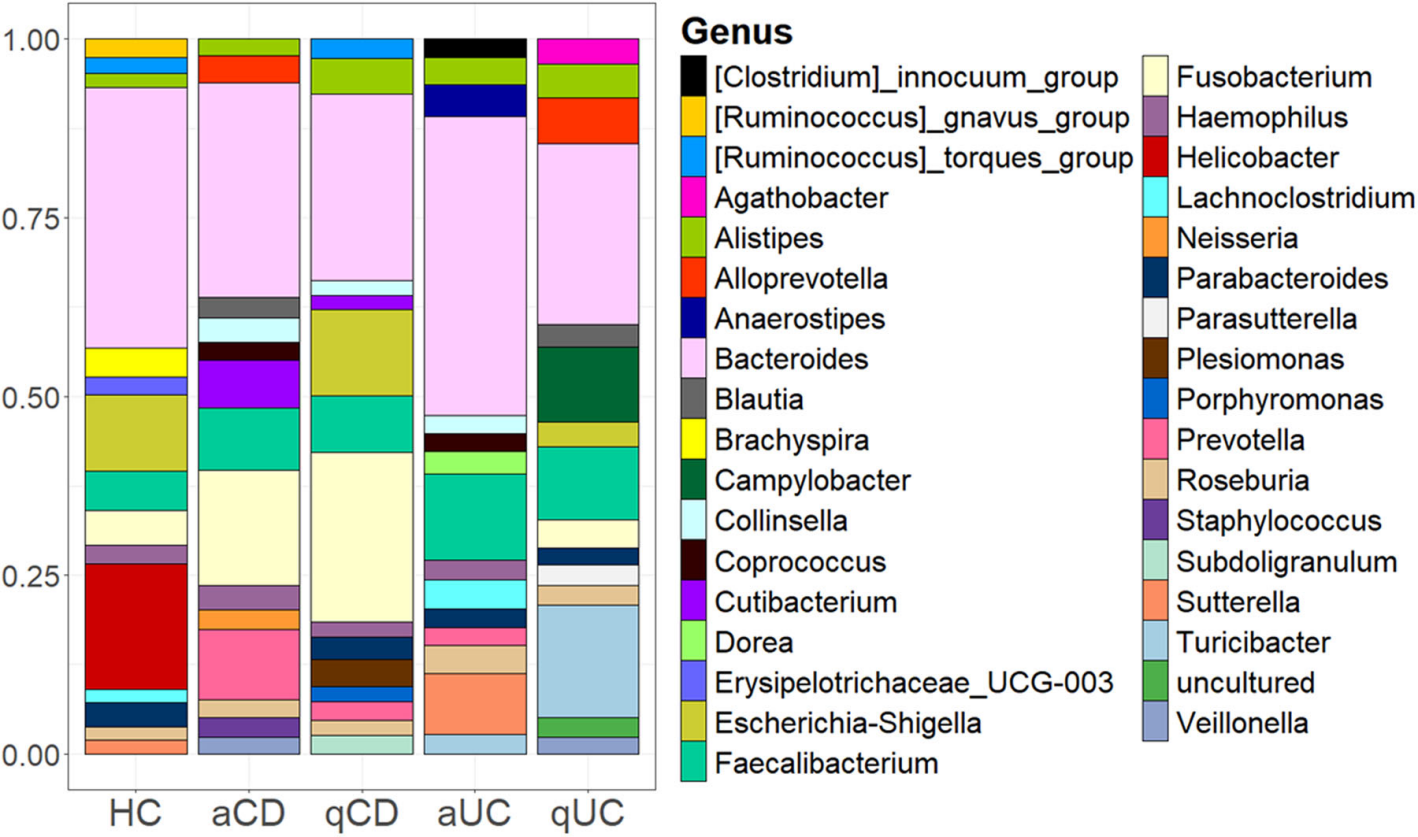
Top 15 genus of bacteria in intestinal biopsies. The 15 most abundant bacteria genera found in intestinal biopsies were identified for each group: healthy controls (HC), active Crohn's disease (aCD), quiescent Crohn's disease (qCD), active colitis (aUC) and quiescent colitis (qUC). Relative frequencies of each genus were calculated and those which relative frequencies were not assigned to any genus (N/A) were discarded. Values of these 15 genera were standardized to 0–1 to calculate the percentage that is represented.

Having analyzed alpha and beta diversities, we assessed the study of bacterial composition. Distinct bacterial signatures were observed among groups (Figure 2). In control group, *Bacteroides*, *Escherichia‐Shigella* and *Helicobacter* were predominant. However, *Helicobacter* was associated only with one patient, so its presence in this group is not representative of a characteristic feature of the control cohort. *Brachyspira*, *Erysipelotrichaceae*, and *Ruminococcus gnavus* were detected exclusively in control samples. Active CD patients exhibited a high abundance of *Bacteroides, Fusobacterium, Prevotella* and *Faecalebacterium*, while in quiescent CD patients, the most abundant genera were *Bacteroides*, *Fusobacterium* and *Escherichia‐Shigella*. Notably *Neisseria* and *Staphylococcus* were exclusive to active CD, while *Porphyromonas*, *Plesiomonas* and *Subdoligranulum* were specific to quiescent CD. *Cutibacterium* was detected in both CD groups, but absent in UC and controls, suggesting its potential association with CD. In patients with active UC the most abundant genera were *Bacteroides*, *Faecalibacterium* and *Sutterella* while *Clostridium innocuum, Dorea*, and *Anaerostipes* were uniquely present in this cohort. Quiescent UC patients showed higher abundance of Bacter*oides, Turicibacter, Faecalebacterium* and *Campylobacter*. *Agathobacter, Campylobacter* and *Parasutterella* were found exclusively in this group. *Turicibacter* was specific to UC and in the same way, *Coprococcus* was identified only in active disease states (both UC and CD).

To further investigate IBD‐associated microbial changes, we compared relative abundance in IBD groups versus controls. The differential abundance analysis performed using LEfSe did not show any significant changes. However, upon examining the Stack bar plots, some trends can be observed. In active CD *Fusobacterium* and *Faecalebacterium* were more abundant, while in quiescent CD, *Fusobacterium* and *Faecalebacterium* and *Alistipes* showed higher abundance. In active UC, there was an increase in *Bacteroides, Faecalebacterium, Lachnoclostridium, Roseburia* and *Sutterella*. Lastly, in quiescent UC *Alistipes* and *Faecalebacterium* were augmented together with a reduction in *Escherichia‐Shigella* compared to controls.

A comprehensive summary of bacterial alterations is described in Table 1.

**Table 1:** Differences in bacterial composition between controls and IBD groups both in biopsies and stool.

Sample	Group	Changes in bacterial genera (increase/decrease)	
Stool	Active CD	↑ Collinsella, *Dialister, Fusobacterium, Parabacteroides, Ruminococcus, Succinivibrio, Prevotella*	↓ *Bacteroides, Anaerostipes, Ruminococcus torques, Erysipelotrchaceae, Lachnoclostridium, Prevotellaceae*
	Quiescent CD	*↑ Ruminococcus gnavus, Catenibacterium, Coprococcus, Enterococcus, Holdemanella, Lactobacillus, Megamonas, Streptococcus*	
Biopsies	Active CD	*↑ Alloprevotella, Neisseria, Staphylococcus, Cutibacterium, Prevotella, Veillonella, ↓ Escherichia‐Shigella*	*↑ Fusobacterium, Faecalibacterium, Alistipes ↓ Brachyspira, Erysipelotrichaceae, Ruminococcus gnavus*
	Quiescent CD	***↑**Alistipes**,** Collinsella, Plesiomonas, Porphyromonas, Prevotella, Subdoligranulum*	
Stool	Active UC	*↑ Christensenellaceae, Coprococcus, Prevotella, Faecalebacterium, Collinsella, Streptococcus*	↓ *Bacteroides, Anaerostipes, Ruminococcus torques, Erysipelotrchaceae, Lachnoclostridium, Prevotellaceae*
	Quiescent UC	↑ *Collinsella, Coprococcus, Escherichia‐Shigella, Roseburia, Subdoligranulum, Streptococcus*	
Biopsies	Active UC	*↑ Bacteroides, Faecalibacterium, Lachnoclostridium, Roseburia, Sutterella, Clostridium innocuum, Dorea, Anaerostipes*	*↑ Faecalibacterium, Alistipes, Turicibacter ↓ Brachyspira, Erysipelotrichaceae, Ruminococcus gnavus*
	Quiescent UC	*↑ Agathobacter, Alloprevotella, Blautia, Campylobacter, Faecalibacterium, Parabacteroides, Parasutterella, Veillonella ↓ Escherichia‐Shigella*	

### 3.2 Fungi Diversity and Composition

**Figure 3:**
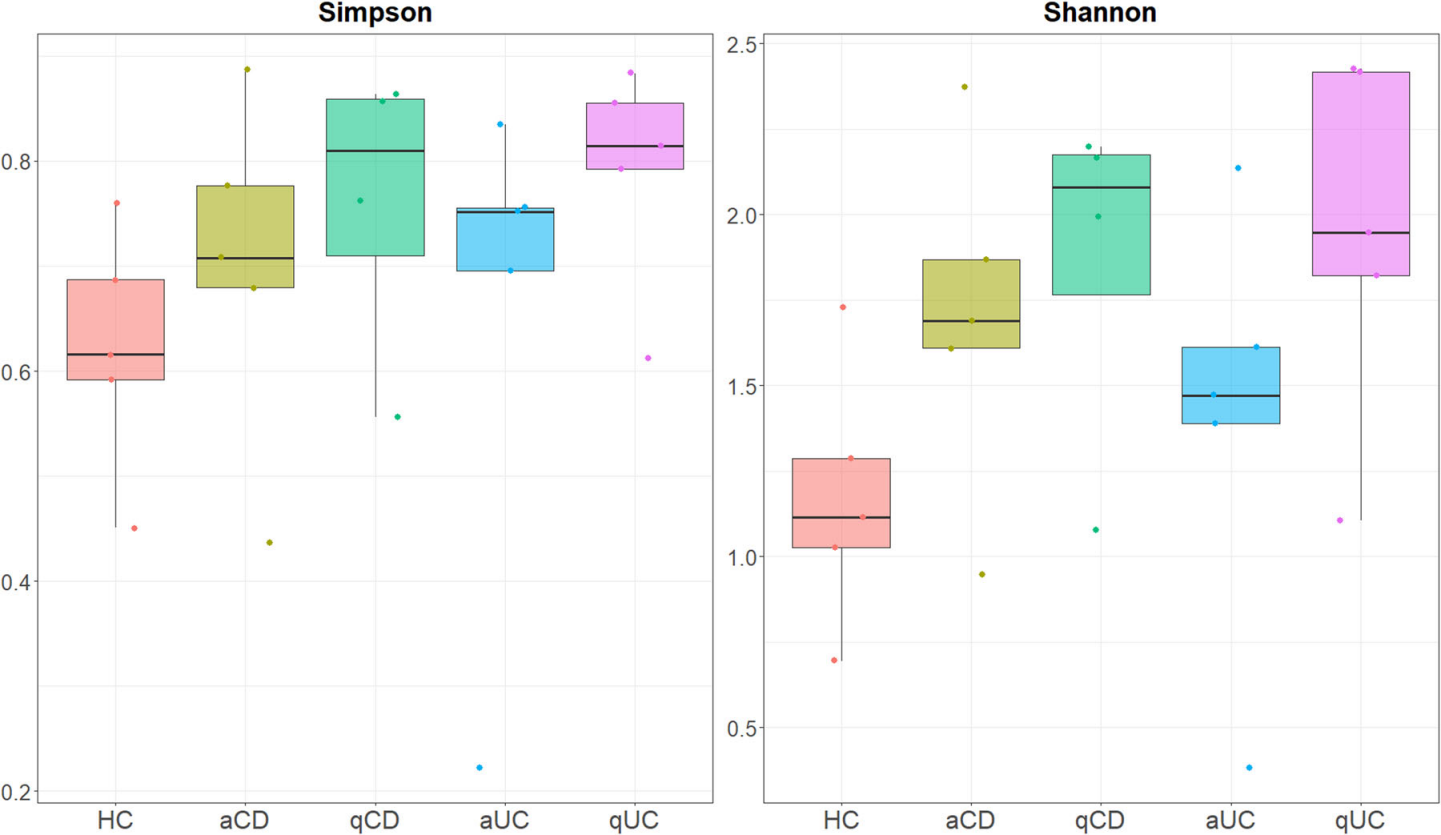
Fungal alpha diversity in intestinal biopsies. Shannon and Simpson indices of alpha diversity of fungus were calculated in intestinal biopsies from the different studied groups: healthy controls (HC), active Crohn's disease (aCD), quiescent Crohn's disease (qCD), active colitis (aUC) and quiescent colitis (qUC). Kruskal Wallis test was then performed. *p*‐value < 0.05 was considered significant.

Bacterial microbiota has been extensively studied in IBD, however, mycobiome remains less explored, despite its potential role in IBD. Alpha diversity was analysed using both Shannon and Simpson indices (Figure 3: alpha_diversity_fungi). Kruskal Wallis test was also performed and no significative differences among groups were observed (Shannon or Simpson index).

The control group tended to have lower fungal diversity, characterized by a moderate number of species and a clear dominance of a few taxa, although without statistical significance. Compared to controls, IBD patients with active disease appeared to present increased mucosal fungal diversity, but this difference was not statistically significant, although a few species remained predominant. Notably, quiescent IBD patients tended to show the highest fungal diversity, suggesting a shift towards a more complex community, although this was not statistically significant.

Beta diversity analysis showed no significant differences among groups. Bray‐Curtis, Jaccard, and UniFrac (both weighted and unweighted) distances were assessed (Supplementary figure 2).

**Figure 4:**
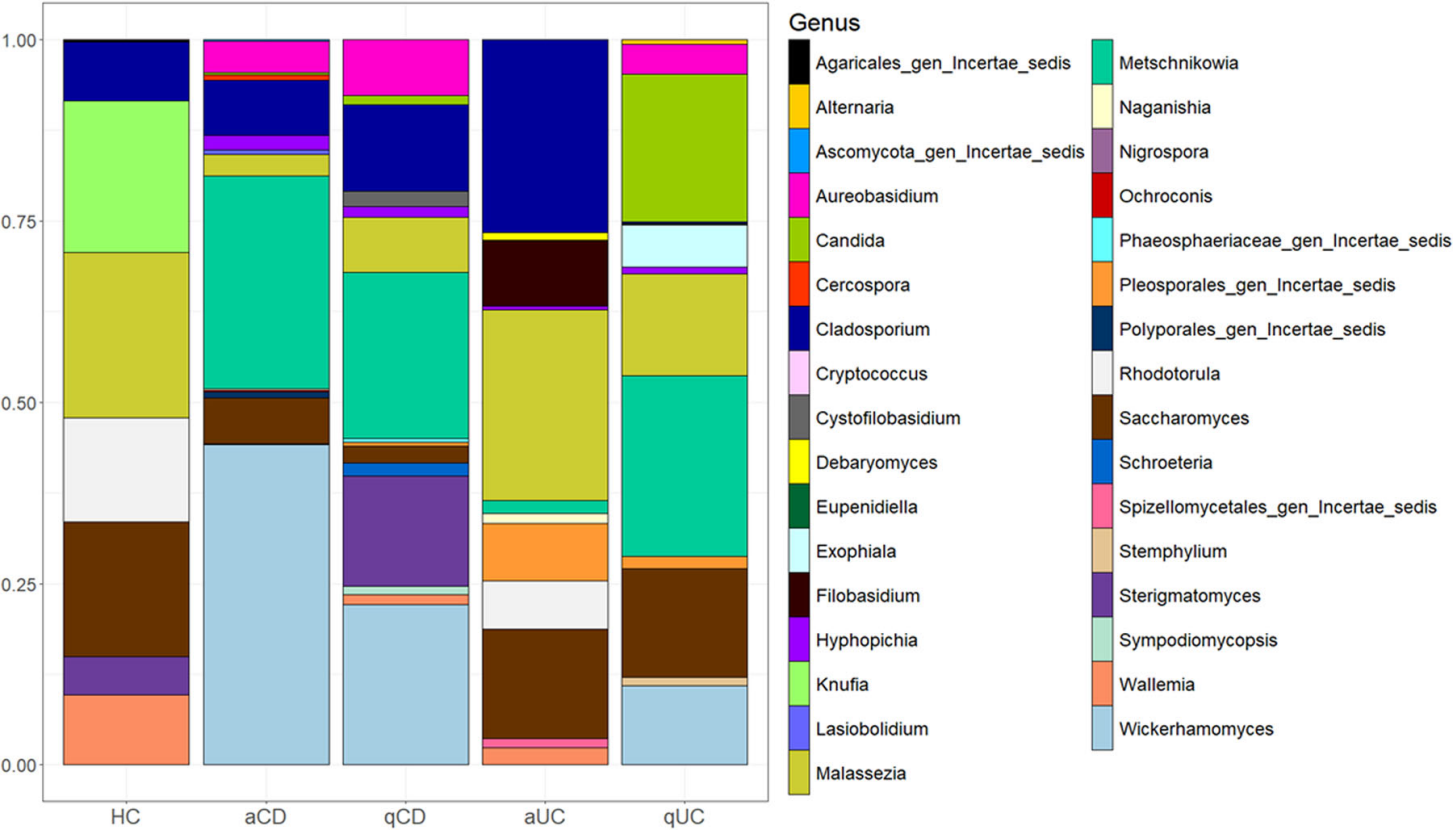
Top 15 genus of fungi in intestinal biopsies. The 15 most abundant fungi genera found in intestinal biopsies were identified for each group: healthy controls (HC), active Crohn's disease (aCD), quiescent Crohn's disease (qCD), active colitis (aUC) and quiescent colitis (qUC). Relative frequencies of each genus were calculated and those which relative frequencies were not assigned to any genus (N/A) were discarded. Values of these 15 genera were standardized to 0–1 to calculate the percentage that is represented.

Having analyzed alpha and beta diversities, we assessed the study of fungi composition. In control samples, a lower diversity of fungal genera was observed compared to IBD cohorts, with only seven genera represented, while IBD groups exhibited between 12 and 15 genera. The genera detected in controls included *Cladosporium*, *Candida*, *Malassezia*, *Rhodotorula*, *Saccharomyces*, *Sterigmatomyces*, and *Wallemia*; however, none were exclusive to this group. Distinct fungal signatures were observed across disease groups. In active CD, *Metschnikowia* and *Wickerhamomyces* were predominant, while *Cercospora*, *Lasiobolidium*, and *Polysporales* were exclusive to this cohort. Similarly, in quiescent CD, *Metschnikowia* and *Wickerhamomyces* remained the most abundant, with *Cystofilobasidium*, *Phaeosphaeriaceae*, *Schroeteria*, and *Sympodiomycopsis* being specific to this group. In active UC, Cladosporium and *Malassezia* were the most abundant genera, with *Debaryomyces*, *Filobasidium*, *Naganishia*, and *Spizellomycetales* uniquely present in this cohort. In quiescent UC, *Metschnikowia* and *Malassezia* were predominant, while *Alternaria*, *Exophiala*, and *Stemphylium* were exclusively found in this group. Although no genus was specifically associated with either UC or CD, nor with disease activity, *Hypopichia* and *Metschnikowia* were identified in all IBD groups but were absent in controls. Additionally, *Aureobasidium* and *Wickerhamomyces* were present in all IBD cohorts except in active UC (Figure 4).

To further investigate fungal alterations in IBD, we visually and exploratorily examined the stacked bar plots, comparing the relative abundance of the top 15 genera between IBD groups and controls. In active UC, *Cladosporium* has higher relative abundance, while *Rhodotorula* has less compared to controls. In quiescent UC, *Cladosporium* and *Malassezia* showed a decrease relative to controls. Lastly, both active and quiescent CD biopsies exhibited a reduction in *Malassezia* and *Saccharomyces* compared to controls.

A summary of all the alterations in fungi genera in biopsies described in the different disease groups are represented in Table 2.

**Table 2:** Fungal alterations in IBD observed in IBD groups with respect to the controls.

Sample	Group	Changes in fungal genera (increase/decrease)	
Biopsies	Active CD	*↑ Cercospora, Lasiobolidium, Polysporales*	*↑ Metschnikowia, Wickerhamomyces ↓Malassezia, Saccharomyces*
	Quiescent CD	*↑ Cystofilobasisium, Phaeosphaeriaceae, Shroeteria and Sympodiomycopsis*	
	Active UC	*↓Rhodotorula*, *↑ Cladosporium, Malassezia, Debaryomyces, Filobasidium, Naganishia, Spizellomycetales*	*↑Pleosporales*
	Quiescent UC	*↓Cladosporium, Malassezia*, *↑ Metschnikowia, Malassezia, Alternaria, Exophiala, Stemphylium*	

#### 3.2.1 Viral Composition

Similar to the mycobiome, the virome remains largely unexplored. In this study we aim to characterize the viral composition in intestinal biopsies across all cohorts. However, the low proportion of viral DNA compared to the host DNA (human) resulted in an insufficient number of reads, limiting a comprehensive analysis. As a result, viral sequences were underrepresented, limiting further analysis.

### 3.3 Microbiota Correlation in Intestinal Biopsies

Microbial interactions within the gut ecosystem were explored through bacterial‐fungal correlation analysis. Spearman's rank correlation coefficient was calculated to analyze associations between controls, active UC, quiescent UC, active CD and quiescent CD (Supporting Information S1: Figure S3). Significant correlations and their principal characteristics in biopsies are summarized in Table 3. The associations identified should be considered as the result of a preliminary study and should be further tested in more in depth studies. For a better comprehension of the role of the most relevant bacterial and fungal genera, Tables 4 and 5 compile their main features and potential effects in health.

**Table 3:** Significant correlations between bacteria and fungi genus (*p*‐value < 0,05).

Group	Fungi	Bacteria	*R*
Control	*Wallemia*	*Fusobacterium*	−0,89
	*Wallemia*	*Roseburia*	0,89
Active UC	*Hyphopichia*	*Prevotellaceae*	1
	*Filobasidium*	*Prevotellaceae*	1
	*Cladosporium*	*Sutterella*	0,89
Quiescent UC	*Stemphylum*	*Turicibacter*	1
	*Mortierellales*	*Turicibacter*	1
	*Eupenidiella*	*Turicibacter*	1
	*Cryptococcus*	*Turicibacter*	1
	*Alternaria*	*Turicibacter*	1
	*Hyphopichia*	*Campylobacter*	0,92
	*Cladosporium*	*Collinsella*	0,92
	*Cladosporium*	*Fusobacterium*	−0,89
	*Candida*	*Blautia*	−0,97
	*Aureobasidium*	*Parabacteroides*	−0,89
	*Aureobasidium*	*Parasutterella*	−0,89
	*Aureobasidium*	*Roseburia*	−0,89
Active CD	*Metschnikowia*	*Roseburia*	1
	*Aureobasidium*	*Collinsella*	−0,89
	*Aureobasidium*	*Roseburia*	−0,89
Quiescent CD	*Sympodiomycopsis*	*Plesiomonas*	1
	*Sterigmatomyces*	*Plesiomonas*	1
	*Pleosporales*	*Erysipelotrichaceae*	1
	*Phaeosphaeriaceae*	*Erysipelotrichaceae*	1
	*Hyphopichia*	*Erysipelotrichaceae*	1

**Table 4:** Principal characteristics of most relevant bacteria genus.

Bacterial genus	Principal characteristics	Health effects	References
*Agathobacter*	✓ Produce butyrate, a short‐chain fatty acid which contributes to energy homeostasis, colonic motility, immunomodulation and suppression of gut inflammation ✓ Involved in the fermentation of carbohydrates ✓ Some strains can utilize a range of dietary and host‐derived carbohydrates	✓ Considered part of the core human gut microbiome ✓ Increased abundance associated with barley consumption, which may have positive effects on glucose tolerance ✓ Less prominent in patients with ulcerative colitis compared to healthy individuals	Goto et al. (2021); Lv et al. (2024)
*Anaerostipes*	✓Butyrate producer ✓Capable of fermenting carbohydrates ✓ Some strains can utilize acetate and lactate to produce butyrate	✓ Considered part of the core human gut microbiome ✓ Some species may be involved in metabolizing the anticancer drug 5‐fluorouracil	Morinaga et al. (2021); Liu et al. (2024)
*Bacteroides*	✓ Play crucial roles in breaking down complex polysaccharides ✓ Capable of degrading and utilizing glycans, including mucin‐type O‐glycans ✓ Produce short‐chain fatty acids (SCFA) as fermentation end products	✓ Maintain a complex and generally beneficial relationship with the host when in the gut ✓ Can become opportunistic pathogens if they escape the gut environment ✓ Associated with bacteraemia and abscess formation in various body sites (*B. fragilis)*	Mukherjee et al. (2020); Wexler (2007)
*Bifidobacterium*	✓ Ferment carbohydrates, producing lactic acid and acetic acid as primary end products ✓ Can break down complex carbohydrates, including oligosaccharides (prebiotics) ✓ Some species can metabolize host‐derived glycans, including mucin	✓ Help maintain gut homeostasis ✓ May provide protection against pathogens through competitive exclusion ✓ Involved in modulating the immune system ✓ May help in mineral absorption and protect against intestinal permeability	Turroni et al. (2019); O'Callaghan and van Sinderen (2016)
*Blautia*	✓ Ferment carbohydrates, producing SCFAs ✓ Can utilize a wide range of carbohydrates, including indigestible ones ✓ Some species can use CO, H_2_/CO_2_, and carbohydrates as energy sources ✓ Produce acetic acid, succinic acid, lactic acid, and ethanol as fermentation end products	✓ Associated with both positive and negative health outcomes ✓ May play a role in alleviating inflammatory and metabolic diseases ✓ Shows antibacterial activity against specific microorganisms ✓ Some species produce health‐promoting compounds like SCFAs and antimicrobial peptides	Maturana and Cárdenas (2021); Liu et al. (2021)
*Clostridium*	✓ Ferment carbohydrates and proteins to produce SCFAs like butyrate ✓ Produce beneficial metabolites like indole propionic acid ✓Metabolize bile acids	✓ Can alter differentiation of T helper 17 cells and regulatory T cells ✓ Many Clostridium species have beneficial effects: help maintain intestinal homeostasis, Strengthen the intestinal barrier and have shown to alleviate colitis and allergic diarrhea ✓ Some species can be pathogenic, for example C. difficile can cause severe diarrhea and colitis	Schirmer et al. (2019); Guo et al. (2020); Martinez et al. (2022)

*Collinsella*	✓ Metabolize bile acids to oxo‐bile acid intermediates	✓ Promotion of inflammation by altering neutrophil chemotaxis and producing an increase in NF‐kB ✓ *Collinsella* abundance was found to be 12‐fold higher in patients with nonalcoholic steatohepatitis (NASH) compared to controls	Astbury et al. (2020)
*Eubacterium hallii*	✓ Produces butyrate from glucose, acetate, and lactate ✓ Capable of utilizing glycerol to produce 3‐hydroxypropionaldehyde (3‐HPA, reuterin) ✓ Converts 1,2‐propanediol to propionate, propanal, and propanol ✓ Produces cobalamin (vitamin B12)	✓ Increases faecal butyrate concentrations ✓ Affects bile acid metabolism, potentially impacting glucose and energy homeostasis	Udayappan et al. (2016); Engels et al. (2016)
*Faecalibacterium*	✓ Major butyrate producer in the gut ✓ Acetate consumer (acetate cross‐feeding) ✓ Ferments indigestible fiber	✓ Considered a biomarker for a healthy gastrointestinal tract ✓ Decreased abundance linked to inflammatory bowel diseases (IBD) and colorectal cancer ✓ Possesses anti‐inflammatory properties ✓ May act as a keystone taxon in stabilizing the gut microbiota	Martín et al. (2023); Fitzgerald et al. (2018)
*Fusobacterium*	✓ Produce butyric acid as a major end product of metabolism ✓ Unable to ferment carbohydrates	✓ Part of the normal flora in the human gut mucosa, it is found particularly in the colon ✓ Implicated in colorectal cancer development ✓ Linked to inflammatory bowel diseases ✓ Can induce secretion of specific IgA antibodies	Quaglio et al. (2022); Tahara et al. (2014); Citron (2002)
*Lactobacillus*	✓ Metabolize carbohydrates to produce lactic acid ✓ Some species can ferment indigestible fibers ✓ Aid in digestion of certain dietary substrates, including lactose	✓ Strengthen intestinal barrier function ✓ Increase mucus production ✓ Stimulate release of antimicrobial peptides ✓ Enhance production of secretory immunoglobulin A (sIgA) ✓ Increase tight junction integrity of intestinal epithelial cells ✓ Provide competitive resistance against pathogens	(Dempsey and Corr (2022); Rastogi and Singh (2022))
*Parabacteroides*	✓ Produce acetic and succinic acids as major degradation products of sugars ✓ Capable of carbohydrate metabolism ✓ Secrete short‐chain fatty acids	✓ Associated with metabolic syndrome, inflammatory bowel disease, and obesity ✓ Some species (*P. distasonis and P. goldsteinii*) show potential as next‐generation probiotics due to protective effects on inflammation and obesity in mice	Cui et al. (2022); Sun et al. (2023)

*Prevotella*	✓ Associated with plant‐rich diets high in complex carbohydrates ✓ Capable of metabolizing various plant polysaccharides	✓ Associated with both beneficial and potentially detrimental effects ✓ Linked to improved glucose metabolism and reduced visceral fat ✓ Also associated with chronic inflammatory conditions, insulin resistance, and hypertension ✓ Found in inflamed tissue in UC patients	Yeoh et al. (2022); Prasoodanan P. K. et al. (2021); Walker et al. (2011)
*Roseburia*	✓ Produce SCFAs, particularly butyrate ✓ Ferment complex polysaccharides ✓ Prefer an acidic intestinal environment	✓ Part of the normal gut microbiota, primarily inhabit the human colon ✓ Produced butyrate serves as an energy source for colonocytes ✓ Helps maintain gut barrier function	Nie et al. (2021); Duncan et al. (2002)
*Ruminococcus gnavus*	✓ Capable of utilizing both dietary carbohydrates and host‐derived sugars ✓ Some strains have evolved to preferentially use sugars found in the gut lining	✓ Associated with both health and disease states ✓ Increased abundance linked to various intestinal disorders (IBD, IBS, colon cancer) ✓ Also associated with extra‐intestinal conditions (skin allergies, cardiovascular diseases, liver diseases, brain disorders)	Bell et al. (2019); Crost et al. (2023)
*Ruminococcus torques*	✓ Capable of degrading mucin glycoproteins and O‐linked glycans ✓ Utilizes both mucin glycoproteins and released oligosaccharides from gastric and colonic mucins ✓ Possesses strong fucosidase, sialidase, and β1,4‐galactosidase activities ✓ Lacks detectable sulfatase activity and has weak β1,3‐galactosidase activity ✓ Secretes a variety of enzymes which are involved in the degradation of mucin and mucin‐derived glycans	✓ Associated with inflammatory bowel diseases (IBDs) in multiple studies ✓ Its mucin‐degrading ability may contribute to defects in mucus protection ✓ Potential target for preventing or treating IBD due to its keystone role in mucin degradation	Schaus et al. (2024)
*Shigella*	✓ Generally, cannot ferment lactose ✓ Produce organic acids from carbohydrate or peptone metabolism	✓ Highly pathogenic, causing shigellosis (bacillary dysentery) ✓ Invade the epithelial lining of the colon, causing severe inflammation and cell death ✓ Trigger release of pro‐inflammatory cytokines (IL‐1β, IL‐18) ✓ Suppress innate immune responses ✓ Interfere with adaptive immune responses, leading to partial susceptibility to re‐infection	Schroeder and Hilbi (2008); Mattock and Blocker (2017)
*Turicibacter*	✓ Produce short‐chain fatty acids, primarily lactate, with smaller amounts of acetate and butyrate	✓ Primarily found in the gut ✓ Different *Turicibacter* strains exhibit varying abilities to modify bile acids ✓ Possess bile salt hydrolases (BSHs), so they may modulate serum bile acid profile ✓ Can influence host lipid and cholesterol metabolism	Lynch et al. (2023); Imamura et al. (2024); Lin et al. (2023)

**Table 5:** Principal characteristics of most relevant fungi genus.

Fungi genus	Principal characteristics	Health effects	References
*Aureobasidium*	✓ They can produce a wide variety of compounds, including enzymes, polysaccharides, and biosurfactants ✓ Some species can ferment to produce β‐polymalic acid, laccase, liamocins, and pullulan polysaccharides	✓ Its presence in the gut would likely be transient or incidental rather than as an established colonize	Rensink et al. (2024); Wu et al. (2023)
*Candida*	✓ *Candida* species can ferment various sugars, with different species having distinct fermentation profiles ✓ They can adapt to different nutrient environments within the gut	✓ It is part of the normal flora of the gastrointestinal tract in many healthy individuals ✓ *Candida* species interact with the host immune system and can modulate immune responses ✓ Overgrowth of *Candida* in the intestine has been linked to various gastrointestinal disorders, including inflammatory bowel disease and irritable bowel syndrome ✓ In mice, can induce protective immune responses against invasive candidiasis, mediated by elevated systemic anti‐*C. albicans* Th17 cells and IL‐17 responsive neutrophils	Shao et al. (2019); Kreulen et al. (2023)
*Cladosporium*	✓ Can produce cladosporide A, an antifungal agent against the human pathogenic filamentous fungus Aspergillus fumigatus	✓ It is a common opportunistic fungus with the ability to colonize the gastrointestinal tract ✓ Over half of the natural products isolated from *Cladosporium* have been found to have various biological activities, including cytotoxic, antibacterial, antiviral, antifungal and enzyme‐inhibitory activities	Gutierrez et al. (2022); Li et al. (2024)
*Exophiala*	✓ *Exophiala* can produce three different types of melanin ✓ *Exophiala* species are polyextremotolerant, able to survive in harsh environments	✓ While not typically associated with the gut, *Exophiala* has been found to colonize the human intestine and respiratory tract ✓ In the gut of UC patients there are less amount of *Exophiala* compared to healthy controls	Beheshti‐Maal et al. (2021); Singh et al. (2021)
*Filobasidium*	✓ *Filobasidium* species can ferment various sugars ✓ They are known to assimilate a wide range of carbon compounds ✓ Some species produce extracellular enzymes that could potentially aid in nutrient acquisition	✓ The enriched presence of *Filobasidium* spp. in donor faeces is associated with the positive response to Faecal microbiota transplantation (FMT) for patients with UC ✓ *Filobasidium* species were found near non‐inflamed tissue in biopsies from Crohn patients	van Thiel et al. (2022); Miyoshi et al. (2018)
*Malassezia*	✓ *Malassezia* species are lipophilic, meaning they require lipids for growth	✓ *Malassezia* is part of the human mycobiome ✓ They can modulate immune responses, particularly the innate immune system ✓ It can interact with pattern‐recognition receptors (PRRs) like Toll‐like receptors (TLRs), leading to the release of cytokines ✓ Higher prevalence of Malassezia in the intestines of patients with Crohn's disease, where it might trigger immune responses contributing to inflammation	Iliev and Leonardi (2017); Underhill and Iliev (2014)
*Rhodotorula*	✓ They produce the enzyme urease and do not ferment carbohydrates ✓ *Rhodotorula* species produce carotenoids	✓ *Rhodotorula* species are commonly found in the human gastrointestinal tract ✓ They may have a probiotic effect by regulating the multiplication of pathogenic bacteria and neutralizing or destroying their toxins ✓ Colonized humans may benefit from nutrients produced by *Rhodotorula*, including proteins, lipids, folate, and carotenoids ✓ Some strains of *Rhodotorula* are being studied for their potential beneficial effects on immune function and gut microbiota	Herbert Hof (2019); Ge et al. (2021)
*Saccharomyces*	✓ *S. cerevisiae* can ferment various sugars ✓ It produces enzymes like urease and does not ferment carbohydrates ✓ Produce antimicrobial peptides, modulate the immune system, and have trophic effects	✓ *Saccharomyces* species can influence the composition of the gut microbiota, potentially increasing beneficial bacteria and reducing harmful ones ✓ *S. boulardii* is widely used as a probiotic for treating gastrointestinal disorders, particularly diarrhea ✓ It can improve gut barrier function ✓ *Saccharomyces* is mostly associated with an anti‐inflammatory effect on dendritic cells, as well as suppression of the exacerbated activation of the NLRP3 inflammasome both in patients and in murine models of IBD	Pais et al. (2020); Sun et al. (2021); Thomas et al. (2011)
*Wallemia*	✓ *Wallemia* species can produce toxins even under saline conditions ✓ They are considered filamentous food‐borne pathogenic fungi	✓ While not primarily considered intestinal fungi, *Wallemia* species can be ingested through contaminated food. ✓ *W. sebi, W. mellicola, and W. muriae* have been reported to be related to human health problems.	Zajc and Gunde‐Cimerman (2018)

### 3.4 Microbial Diversity in Faeces

#### 3.4.1 Bacterial Diversity and Composition

Bacterial communities in the human microbiota have typically been studied either in faecal samples or intestinal biopsies separately. To provide a more comprehensive view of the human bacteriome, bacterial composition of the faeces of the same patients whose mucosal samples had been previously examined, was analyzed.

**Figure 5:**
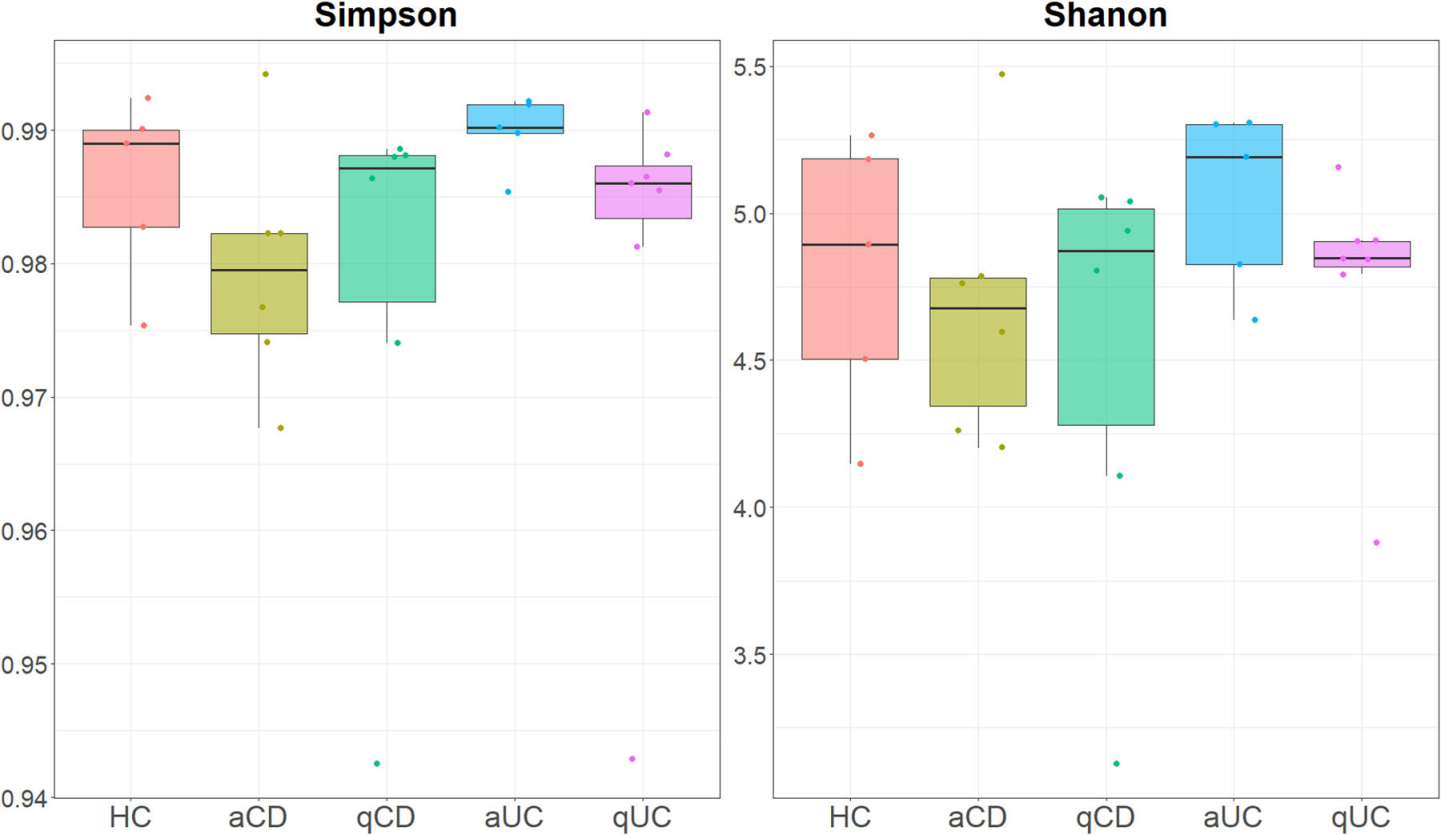
Bacterial alpha diversity in stool samples. Shannon and Simpson indices of alpha diversity of the bacteria were calculated in stool samples from the different studied groups: healthy controls (HC), active Crohn's disease (aCD), quiescent Crohn's disease (qCD), active colitis (aUC) and quiescent colitis (qUC). Kruskal Wallis test was then performed. *p*‐value < 0.05 was considered significant.

First, to assess bacterial diversity in intestinal biopsies, alpha diversity was analyzed using both Shannon and Simpson indexes (Figure 5). Bacterial alpha diversity from faecal samples—as in microbial samples from biopsies—did not differ (Figure 5).

Alpha diversity was very similar between groups (as indicated similar values in both Shannon and Simpson indices), however in controls, Shannon and Simpson values showed greater microbial diversity with a balanced microbiome and no dominance by specific species while a reduction in microbial diversity was observed during the active phases of CD and UC. Finally, in both quiescent CD and UC samples a little recovery of the dysbiosis with no dominance by any specie was observed.

Beta diversity was evaluated using Bray Curtis, Jaccard and Unifrac (weighted and unweighted) distances metrics. No significant differences were observed between groups (Supporting Information S1: Figure S4).

**Figure 6:**
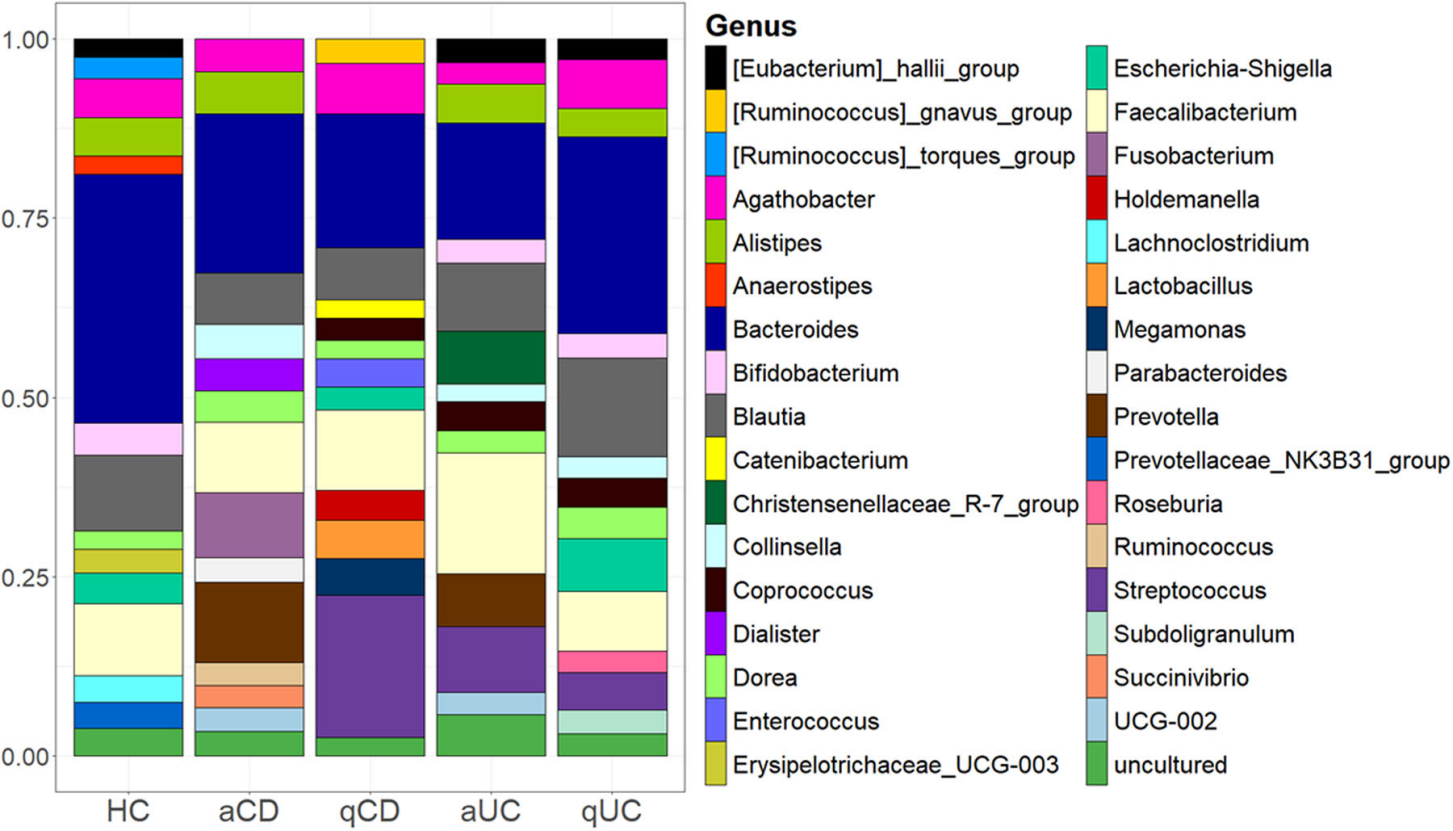
Top 15 genus of bacteria in stool samples. The 15 most abundant bacteria genera found in stool samples were identified for each group: healthy controls (HC), active Crohn's disease (aCD), quiescent Crohn's disease (qCD), active colitis (aUC) and quiescent colitis (qUC). Relative frequencies of each genus were calculated and those which relative frequencies were not assigned to any genus (N/A) were discarded. Values of these 15 genera were standardized to 0–1 to calculate the percentage that is represented.

Having studied alpha and beta diversities, we also assessed the analysis of bacterial composition in faeces. Our results showed that *Bacteroides*, *Blautia*, and *Faecalibacterium* were the most prevalent genera, with *Streptococcus* also being highly represented in quiescent CD stool samples. Distinct bacterial signatures were observed across groups. In controls, five genera were exclusively identified: *Ruminococcus torques, Anaerostipes, Erysipelotrichaceae, Lachnoclostridium*, and *Prevotellaceae*. In active CD, *Dialister, Fusobacterium, Parabacteroides*, *Ruminococcus*, and *Succinivibrio* were uniquely present, while quiescent CD samples exhibited five specific genera: *Ruminococcus gnavus*, *Catenibacterium*, *Enterococcus*, *Holdemanella*, Lactobacillus, and *Megamonas*. UC stools had fewer exclusive genera, with *Christensenellaceae* found only in active UC and *Roseburia* and *Subdoligranulum* restricted to quiescent UC. Notably, Prevotella was detected solely in active disease states, both in CD and UC (Figure 6).

To further explore bacterial alterations in IBD, differences in the abundance of representative genera between IBD groups and controls were analyzed using LEfSe. Despite these results not showing statistically significant changes between groups, a visual inspection of the stacked bar plots revealed some trends between groups. Stool samples showed greater similarity among cohorts and fewer variations in genus abundance. The most striking change was a decrease in *Bacteroides* across all IBD groups compared to controls. Additionally, *Coprococcus* and *Streptococcus* were present in all IBD groups except active CD, while *Collinsella* was detected in all IBD cohorts except quiescent CD.

A summary of all the alterations in bacterial genera, both in faeces and biopsies described in the different disease groups are found in Table 1.

### 3.5 Microbiota Comparison Between Different Groups

To assess differences between groups (controls, active and quiescent UC, active and quiescent CD) PERMANOVA (Permutational Multivariate Analysis of Variance) was performed in a pairwise manner. PERMANOVA was conducted comparing two groups each time using Bray Curtis distances and was repeated for each analysis (16S faeces, 16S biopsies and ITS2 biopsies). Results are summarized in Supporting Information S2: Table S2. There were only significative differences in fungi diversity between control vs active CD and active UC vs active CD, pseudo‐F value in both comparisons is high (3,6402 and 3,836 respectively) indicating that the variability between groups was significantly greater than the variability within groups.

To analyze the similarities among the different groups ANOSIM (analysis of similarities) was performed in a pairwise manner. ANOSIM was done comparing two groups each time using Bray Curtis distances and was repeated for each analysis (16S faeces, 16S biopsies and ITS2 biopsies). Results are summarized in Supporting Information S2: Table S3. There were significative differences in fungi diversity between control vs active CD and active UC vs active CD and in two cases the statistic R value was relatively close to 1 (0.49 and 0.496, respectively), which meant that differences between groups were significantly greater than differences within groups, according to PERMANOVA results. The ANOSIM test also found significative differences when comparing quiescent UC versus active CD in bacterial analysis in stool.

### 3.6 Microbiota Comparison Between Different Tissues

PERMANOVA was conducted comparing faeces and biopsies in each group using Bray Curtis distances. Results are summarized in Table 6. The PERMANOVA test showed that faeces and biopsies have different compositions in all groups. High Pseudo‐F values (1.5 or higher) in all groups indicated that there were great differences between tissues except in the case of quiescent CD.

**Table 6:** PERMANOVA tests between stool and biopsies in all groups using Bray Curtis distances from bacterial data.

PERMANOVA	Bacteria–Biopsies versus stool	
Group	Pseudo‐F	*p* value
Control	1.7973	0.045*
Active colitis	2.3399	0.016*
Quiescent colitis	1.9253	0.004*
Active Crohn	1.5964	0.036*
Quiescent Crohn	1.4691	0.1

ANOSIM test was conducted between faeces and biopsies in each group using Bray Curtis distances. Results are summarized in Table 7. There were significative differences in controls and both active and quiescent UC groups when comparing stools to biopsies. Statistic R value was relatively close to 1 in both colitis groups (0.41 and 0.48, respectively), which meant that differences between tissues were significantly greater than differences within tissues. This R value was very close to 0 in the active CD group, indicating that the difference between tissues was small and not significant.

**Table 7:** ANOSIM tests between stool and biopsies in all groups using Bray Curtis distances from bacterial data.

ANOSIM	Bacteria–Biopsies versus stool	
Group	Statistic R	*p* value
Control	0.28	0.042*
Active colitis	0.412	0.015*
Quiescent colitis	0.4839	0.002*
Active Crohn	0.088	0.189
Quiescent Crohn	0.0635	0.263

Analyzing the differences and similarities at the taxonomy level between tissues (Figures 2 and 6), some genera were represented in both tissues in several groups, as *Collinsella* and *Prevotella*, both present in active status of IBD diseases. In a similar way, *Escherichia‐shigella* was represented in controls and quiescent phases of IBD, both in biopsies and stools. *Bacteroides* and *Faecalebacterium* were present in all cohorts both in stool and mucosal samples. It is also interesting that *Parabacteroides* were found in mucosal samples from all cohorts except active CD, although in faeces it was found only in active CD group. *Alistipes* genus was identified in the mucosal samples from all cohorts while in faeces was only absent in quiescent CD. *Erysipelotrichaceae_UCG_003* however was present in both tissues also but only in the control groups. Parallelly, some genera were only found in one tissue as *Eubacterium_hallii* and *Bifidobacterium*, only present in faeces, or *Turicibacter* or *Veillonella*, only present in intestinal biopsies.

## 4 Discussion

In this study, we analysed both bacterial and fungal populations in paired intestinal tissue and faecal samples from healthy donors and patients with CD and UC, across active and quiescent phases. Using 16S, ITS and shotgun sequencing, we identified microbial signatures associated with disease activity. Notably, *Prevotella* correlated with active disease, *Fusobacterium* with active CD, and *Roseburia* with remission in UC. Inter‐kingdom exploratory correlations highlighted the potential regulatory role of *Wallemia* in microbial balance. A key finding was the identification in faeces of potential biomarkers, such as *Prevotella* for active disease and *Roseburia* for remission states which offers exciting opportunities for developing less invasive diagnostic tools and improving patient management.

Our results showed a decrease in SCFA producers in both CD and UC which has also been reported by other studies (Andoh and Nishida 2023; Kim et al. 2024) and involves a reduction in the production of butyrate, propionic acid among others that promotes a tolerogenic environment in the gut (Tian et al. 2024; Cummings et al. 1987). IBD involves an increase in other SCFA producing bacteria like *Turicibacter*, *Faecalebacterium*, *Alistipes* confirmed by other authors (Clooney et al. 2021; Zhang et al. 2019; Santoru et al. 2018). One of their primary roles is the production of SCFAs, particularly *Faecalibacterium*, a butyrate‐producer. While *Turicibacter* and *Alistipes* also produce SCFAs, they may produce different types (lactate and propionic acid, respectively) or amounts of them. Indeed, *Alistipes* has been linked to both protective and pathogenic effects as it showed protective benefits against colitis but has also been associated with colorectal cancer development (Martín et al. 2023; Fitzgerald et al. 2018; Parker et al. 2020; Lynch et al. 2023; Imamura et al. 2024; Lin et al. 2023). The depletion of SCFA producing bacteria observed in IBD patients is associated with aberrant immune responses and impaired intestinal barrier integrity (Lavelle and Sokol 2020). Analysis of the fungal diversity showed an increase in the fungi diversity of IBD patients compared with controls. This increase has been observed by other groups (Ott et al. 2008). We hypothesize that the bacterial dysbiosis in IBD facilitates fungal colonization, a process not observed in healthy individuals, as has been supported by studies showing an increase in the relative abundance of *Candida albicans* and a decrease in *Saccharomyces cerevisiae* in IBD patients compared to controls (Sokol et al. 2017; Jawhara 2022). Furthermore, increased fungal diversity in inflamed mucosa of CD patients has been correlated with disease activity and higher levels of pro‐inflammatory cytokines, such as TNF‐α and IFN‐γ (Li et al. 2014).

These findings suggest that IBD‐related dysbiosis creates an environment that facilitates fungal colonization, although further research is necessary to fully elucidate the fungal dysbiosis associated to IBD and its impact on the pathogenesis. Regarding to that, “Our data reveal a significant association between *Wallemia* abundance and the relative presence of potentially beneficial and pathogenic bacteria such as *Roseburia* and *Fusobacterium*.” It is interesting the fact that SCFA producers like *Turicibacter*, *Roseburia* or *Erysipelotrichaceae* were detected in the same mucosal sites as potentially pathogenic fungi like *Stemphylum*, *Cladosporium* or *Aureobasidium* in remission states of the disease—both UC and CD—, indicating a mechanism of regulation of the fungi and bacteria as it has been described in other fungi (Gutierrez et al. 2022). Some authors confirmed that SCFAs, such as acetate, propionate, and butyrate, produced by commensal intestinal bacteria, directly modulate the presence and behaviour of intestinal fungi, particularly *Candida albicans*, inhibiting its growth and its ability to form hyphae (a more invasive form) (Pande et al. 2013; Tso et al. 2018). This regulation is crucial for maintaining fungal eubiosis and preventing dysbiosis associated with inflammation or disease (Gutierrez et al. 2022). Our results support the idea that the mycobiome and bacteriome exhibit mutual regulatory interactions. In summary, while established microbial interactions contribute to shaping the mycobiome, the metabolic complexity of the gut microbiota likely involves additional regulatory mechanisms.

Bacterial biomarkers represent an established tool for patient classification and serve as a less invasive alternative to traditional procedures such as colonoscopy (Pflughoeft et al. 2019; Mohan and Harikrishna 2015), offering a more patient‐friendly approach. To identify these biomarkers in our IBD patients, we assessed bacterial genera that were present in both stool and biopsy samples within the same patient groups (Figures 2 and 6). In this way, *Prevotella* was enriched in IBD patients. While *Prevotella* has been previously described as a biomarker of carbohydrate‐rich diets, its association with IBD in our cohort may reflect the interplay between dietary habits and disease‐related dysbiosis, rather than a disease‐specific biomarker per se. However, we believe our *Prevotella* findings warrant consideration as potential IBD biomarkers, because alterations in IBD patients can persist even after dietary modifications, suggesting that disease‐intrinsic factors contribute to these changes. Moreover, the chronic inflammatory state in IBD may create a microenvironment that perpetuates and stabilizes these bacterial alterations (Yeoh et al. 2022; Prasoodanan P. K. et al. 2021; Walker et al. 2011). *Fusobacterium* was identified as a potential biomarker for active CD and it was also linked with colorectal cancer and IBD (Quaglio et al. 2022; Tahara et al. 2014; Citron 2002) and *Roseburia*, a well‐known SCFA producer related to normal microbiota (Nie et al. 2021; Duncan et al. 2002), for quiescent UC. Summarizing, our results justify further investigation of these genera as potential biomarkers of IBD.

Regarding the limitations of our study, in addition to the low number of samples in each cohort, diet represents another limitation as it is uncontrolled. We acknowledge that diet represent a significant factor influencing gut microbiota composition. Diet‐microbiota interactions are well‐established, with specific dietary patterns known to selectively promote or inhibit particular microbial taxa. However, given the exploratory nature and limited scope of our study, we opted to focus resources on microbial profiling rather than dietary assessment. While this represents a limitation of our work, we believe our findings provide valuable preliminary insights that warrant validation in larger, more comprehensive studies incorporating detailed dietary evaluation.

Our results add to the evidence that bacterial–fungal interactions may be altered in the gut microbiota of patients with IBD, supporting the need for further studies to clarify their role in disease pathogenesis. Despite limitations as the sample size and the inability to characterize the virome, we have proposed three bacteria genera that could act as biomarkers of IBD.

## Author Contributions

**Elisa Arribas‐Rodríguez:** investigation, writing – original draft, methodology, formal analysis, data curation. **María García‐Pizarro:** methodology, software, formal analysis, data curation. **Ángel De Prado:** investigation, methodology, resources. **María Santiago‐Carretero:** software, formal analysis, methodology. **Marta Hernández:** investigation, resources, methodology. **Benito Velayos Jiménez:** resources, methodology, investigation. **Javier García‐Alonso:** resources, methodology, investigation. **Jesús Barrio:** investigation, resources. **José María Eiros:** conceptualization, investigation. **Eduardo Arranz:** conceptualization, investigation, resources. **David Bernardo:** conceptualization, investigation, project administration, resources. **Luis Fernández‐Salazar:** conceptualization, investigation, funding acquisition, validation, visualization, project administration, supervision, resources. **Sara Cuesta‐Sancho:** conceptualization, investigation, funding acquisition, validation, writing – review and editing, project administration, supervision, resources.

## Ethics Statement

All participants provided signed informed consent (approval code by the CEIm Area del Salud de Valladolid PI 22‐2869).

## Conflicts of Interest

The authors declare no conflicts of interest.

## Use of AI Statement

Generative artificial intelligence (ChatGPT GPT‐4o mini) has been used for spelling correction and style enhancement in some paragraphs. All content has been critically reviewed and validated by the author.

## Supporting information


**Supporting Figure 1:** Bacterial beta diversity in intestinal biopsies. **Supporting Figure 2:** Fungal beta diversity in intestinal biopsies. **Supporting Figure 3:** Correlations between bacteria and fungi genus in biopsies. **Supporting Figure 4:** Bacterial beta diversity in stool samples.

**Supplementary Table 1:** Patient demographics. **Supplementary Table 2:** PERMANOVA tests among all groups. **Supplementary Table 3:** ANOSIM tests among all groups.

## Data Availability

The data that support the findings of this study are available from the corresponding author, SCS or EAR, upon reasonable request.
