# Supplementary material for: Fungal‐Bacterial Dysbiosis in IBD: Microbial Biomarkers of Disease Activity
Source: Microbiologyopen. 2025 Nov 4;14(6):e70088. doi: 10.1002/mbo3.70088 (PMC12586356; doi:10.1002/mbo3.70088)

**SUPPLEMENTARY FIGURE LEGENDS**

**Supplementary Figure 1. Bacterial beta diversity in intestinal biopsies.**

PCoA showing beta diversity was calculated using different distances: A) Bray Curtis, B) Jaccard, C) Unweighted Unifrac, D) Weighted Unifrac.


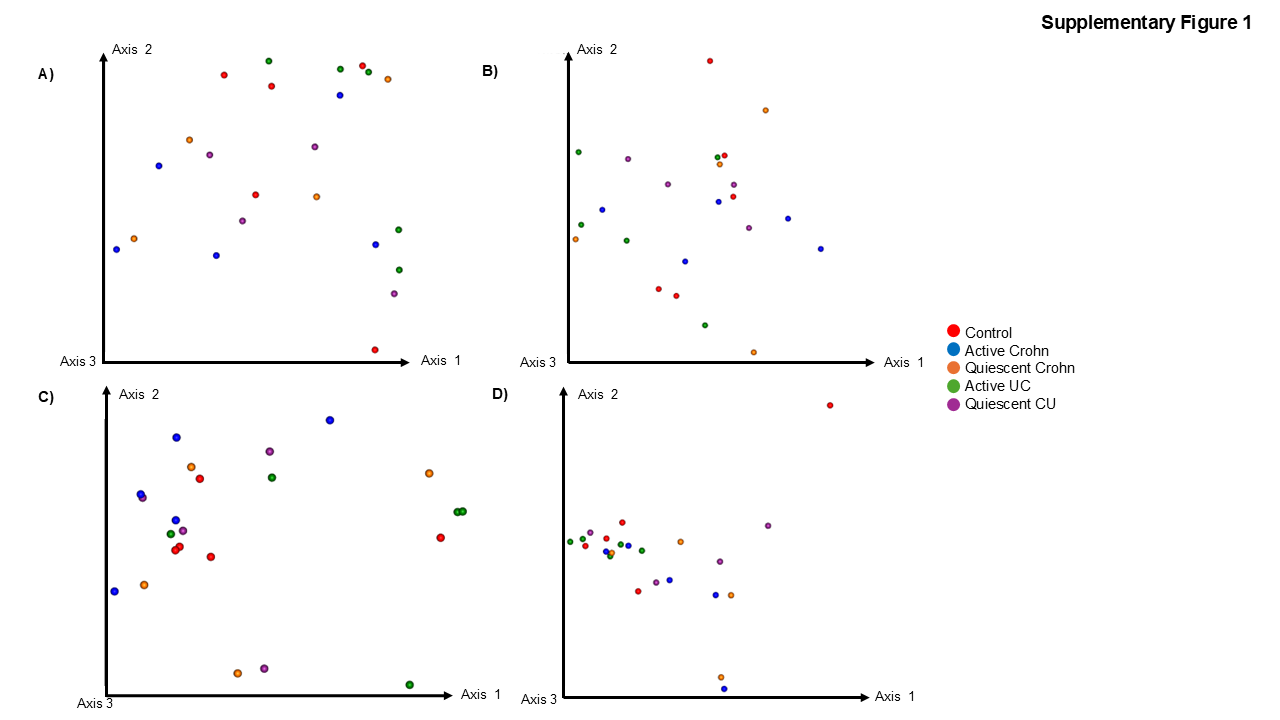


**Supplementary Figure 2. Fungal beta diversity in intestinal biopsies.**

PCoA showing beta diversity was calculated using different distances: A) Bray Curtis, B) Jaccard, C) Unweighted Unifrac, D) Weighted Unifrac.


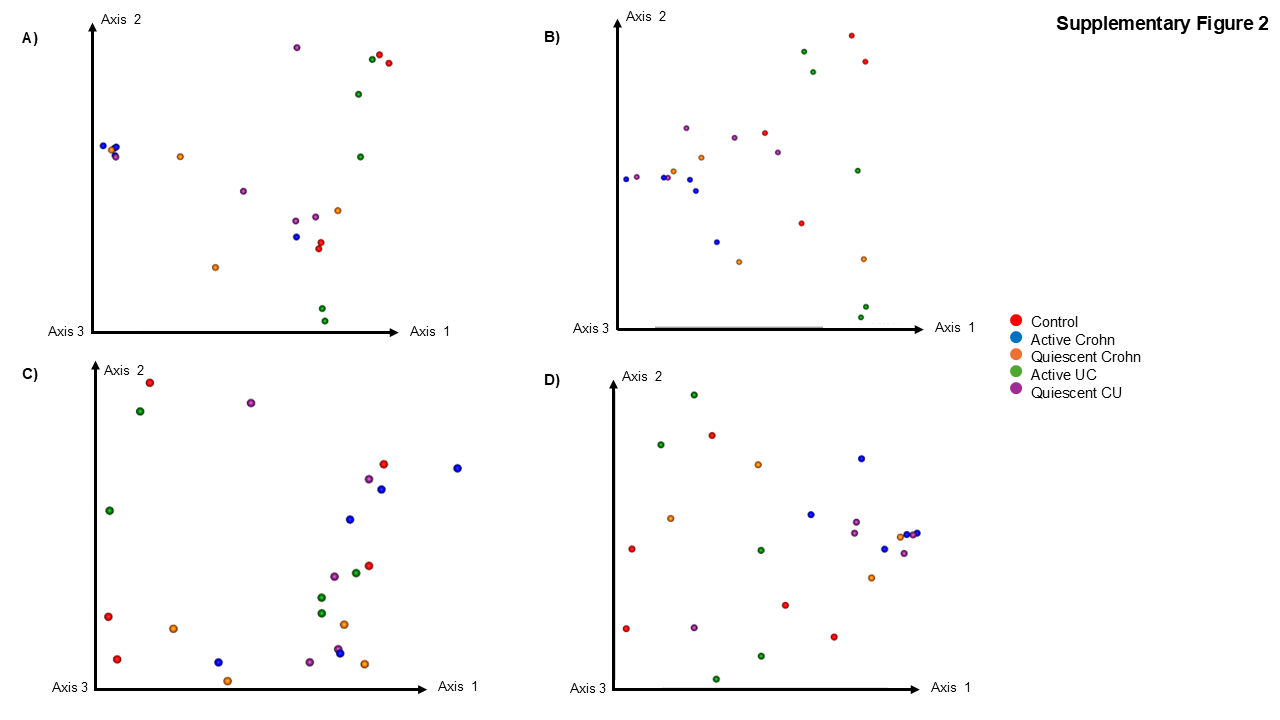


**Supplementary Figure 3. Correlations between bacteria and fungi genus in biopsies.**

Spearman’s rank correlation coefficient was calculated to analyse the correlations between bacteria and fungi in biopsies. Genus which a value of 0 in their relative frequency for all the patients were deleted. The hypothesis of no correlation was checked based on the asymptotic t-student to identify significant correlations. Significant correlations (p-valor < 0,05) are highlighted in bold. A) Controls, B) Active Crohn, C) Quiescent Crohn, D) Active colitis, E) Quiescent colitis.


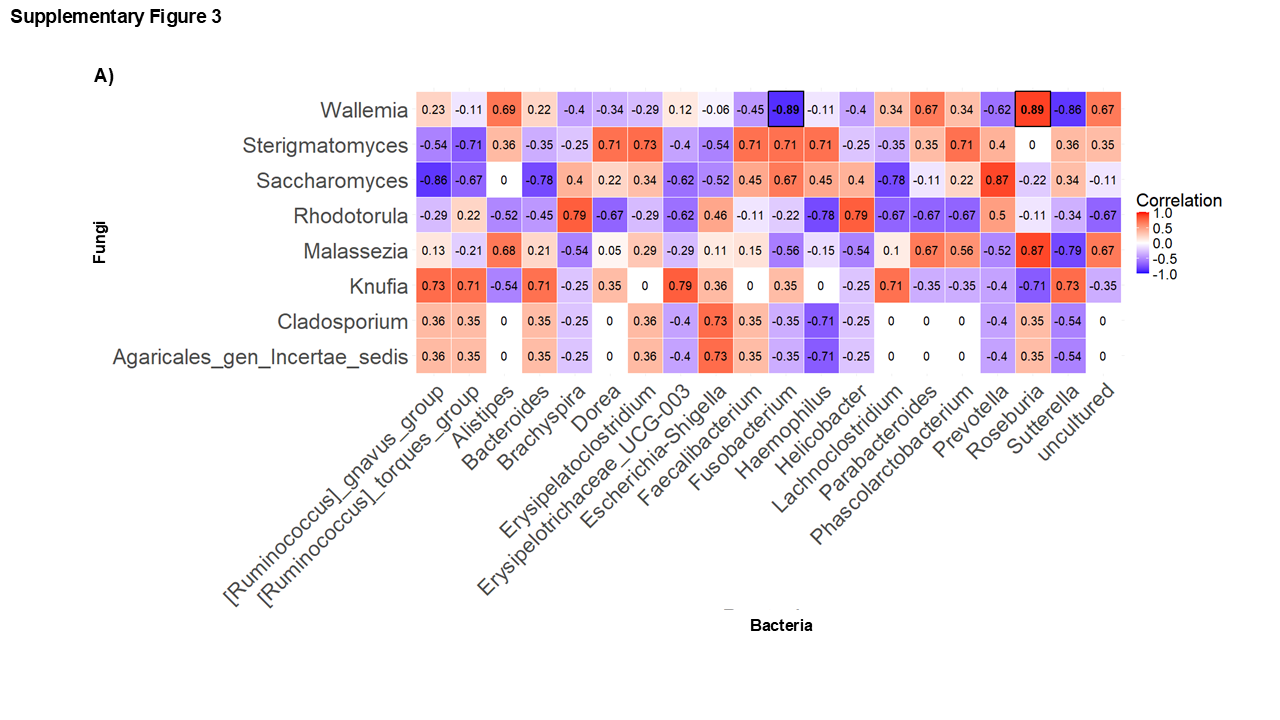


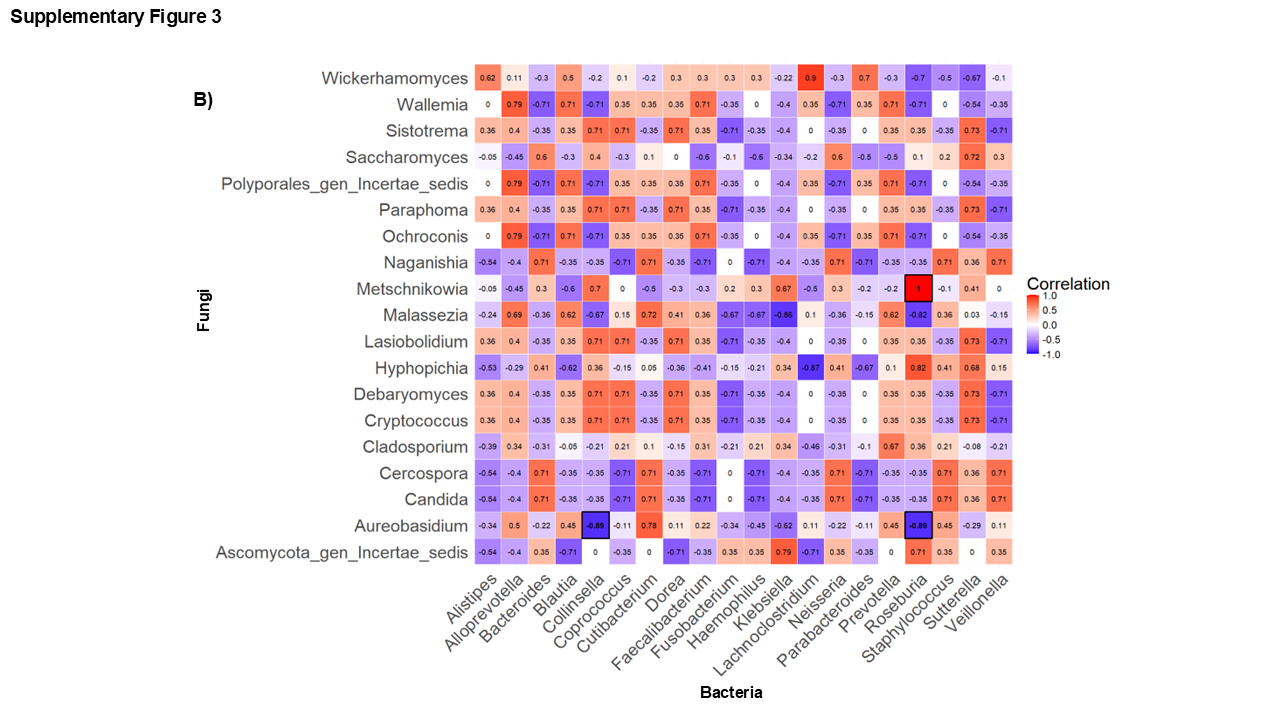


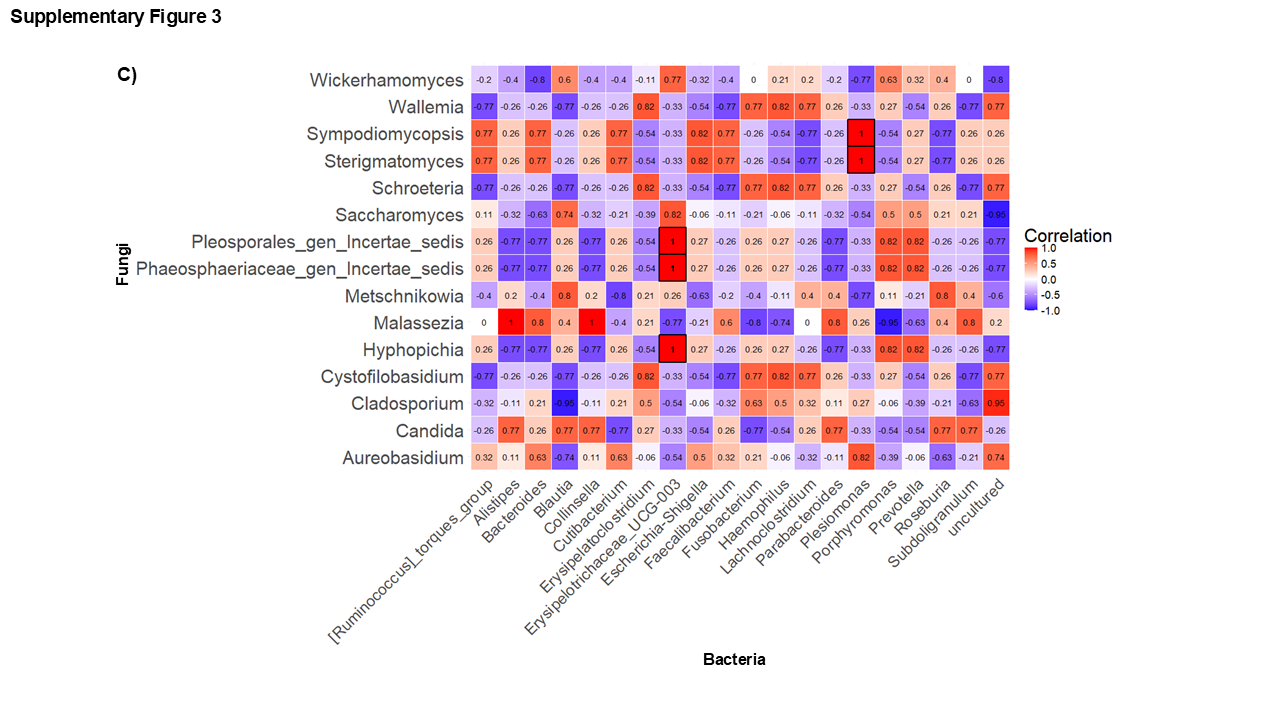


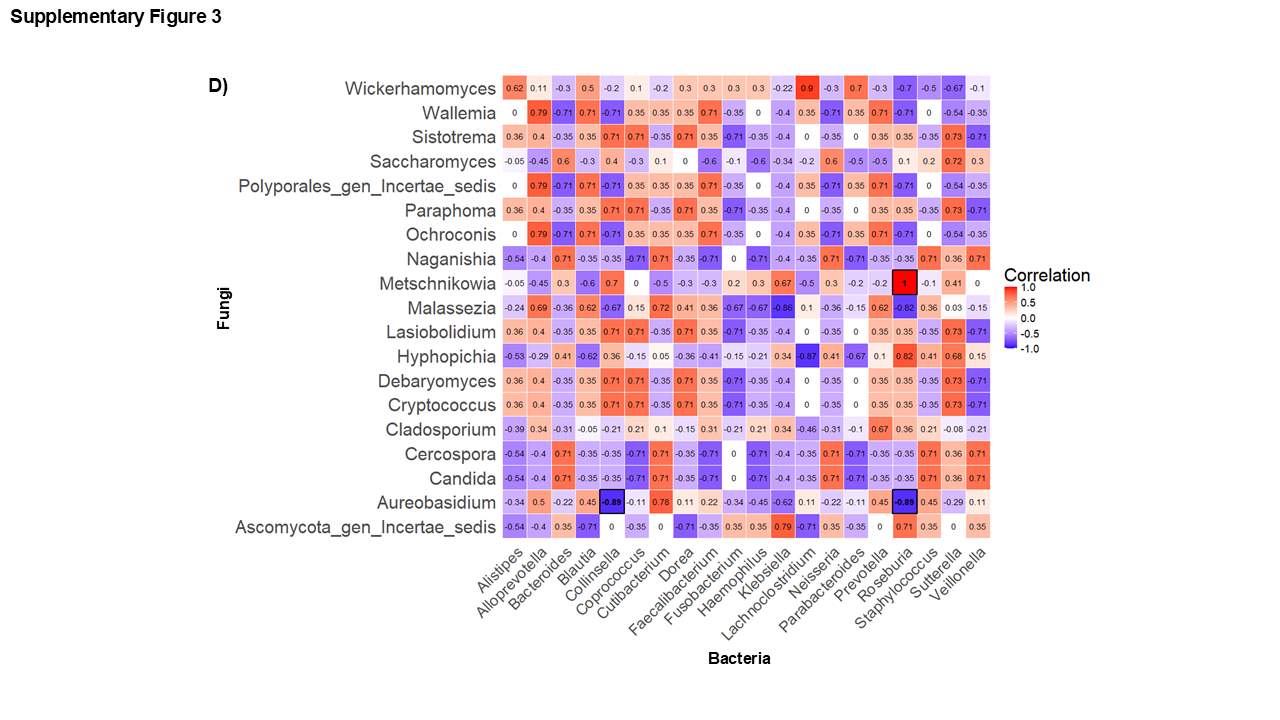


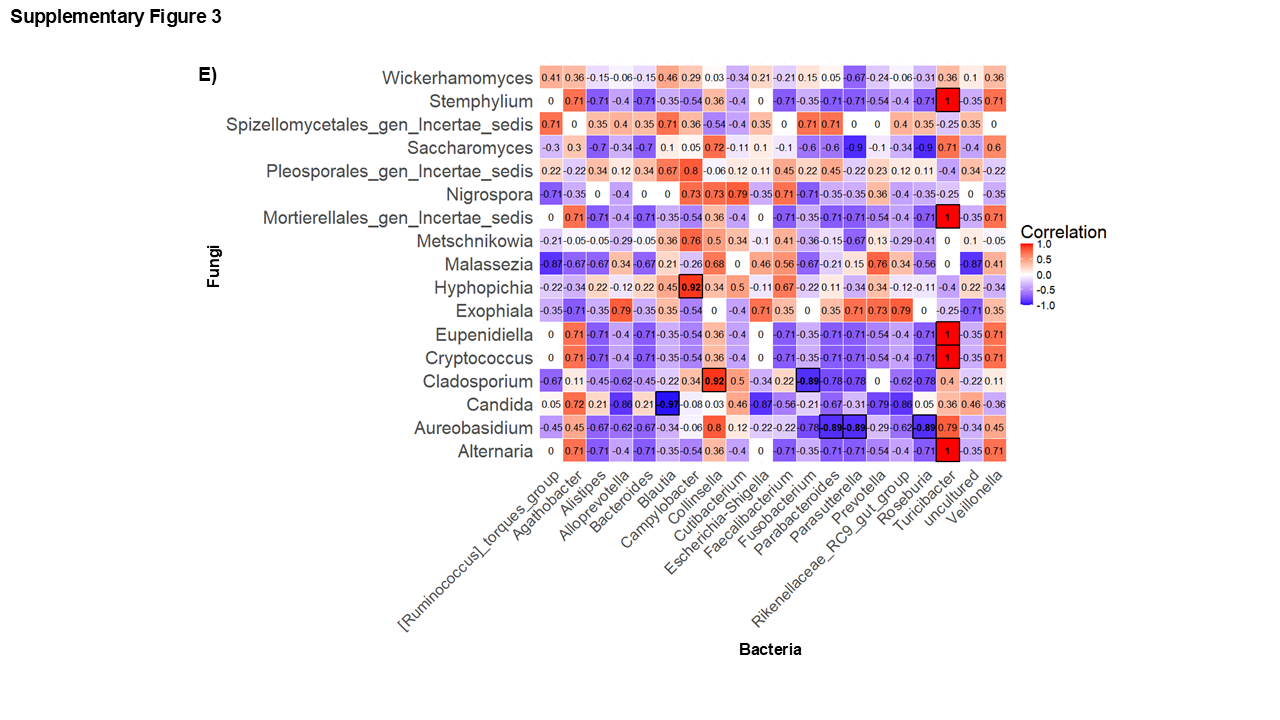


**Supplementary Figure 4. Bacterial beta diversity in stool samples.**

PCoA showing beta diversity was calculated using different distances: A) Bray Curtis, B) Jaccard, C) Unweighted Unifrac, D) Weighted Unifrac.


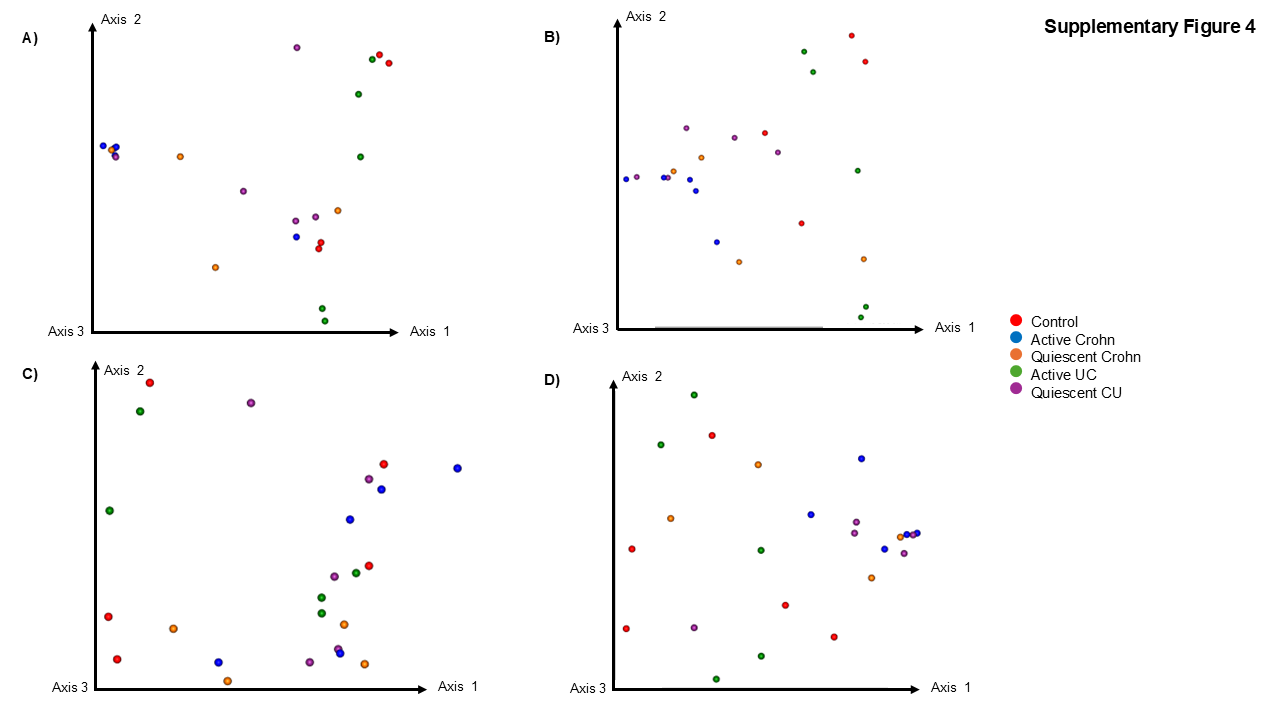

Supplement: Supplementary file 1 — Supporting Figure 1: Bacterial beta diversity in intestinal biopsies. Supporting Figure 2: Fungal beta diversity in intestinal biopsies. Supporting Figure 3: Correlations between bacteria and fungi genus in biopsies. Supporting Figure 4: Bacterial beta diversity in stool samples. [file MBO3-14-e70088-s002.docx]
